# Acute phase proteins and IP-10 in plasma for tuberculosis diagnosis

**DOI:** 10.3389/ftubr.2023.1267221

**Published:** 2023-11-29

**Authors:** Bárbara Molina-Moya, Raquel Villar-Hernández, Nelly Ciobanu, Beatriz Muriel-Moreno, Alicia Lacoma, Alexandru Codreanu, Irene Latorre, Daria Smalchuk, Cristina Prat-Aymerich, Valeriu Crudu, Konstantina Kontogianni, Luis E. Cuevas, José Domínguez

**Affiliations:** ^1^Institut d'Investigació Germans Trias i Pujol, Hospital Universitari Germans Trias i Pujol, CIBER de Enfermedades Respiratorias (CIBERES), Universitat Autònoma de Barcelona, Barcelona, Spain; ^2^Institutia Medico-Sanitara Publica, Institutul de Ftiziopneumologie “Chiril Draganiuc”, Chişinău, Moldova; ^3^Department of Microbiology, Virology and Biotechnology, Odessa National I.I. Mechnikov University, Odessa, Ukraine; ^4^Centre for Drugs and Diagnostics, Department of Clinical Sciences, Liverpool School of Tropical Medicine, Liverpool, United Kingdom

**Keywords:** C-reactive protein, IP-10, α1-acid glycoprotein, α1-antitrypsin, triage

## Abstract

**Background:**

Tuberculosis (TB) is a leading cause of death from a single infectious agent, and triage tests based on biomarkers may help to improve the diagnosis. This study aims to determine whether C-reactive protein (CRP), interferon-γ-inducible protein 10 (IP-10), α1-acid glycoprotein (AGP), and α1-anti-trypsin (AAT) could be useful for a screening test in patients with presumptive TB disease.

**Methods:**

CRP, IP-10, AGP, and AAT were measured in plasma samples from 277 patients with presumptive TB disease in the Republic of Moldova in a prospective study.

**Results:**

In general, the levels of all the biomarkers were higher in patients with TB than in the other groups (*p* < 0.05). Receiver operating characteristic curve analyses showed an area under the curve lower than 0.7 for all the biomarkers, and low correlations (Spearman's *r* < 0.6) were found between biomarkers.

**Conclusion:**

The levels of the tested biomarkers were different throughout the patient groups studied, but their suboptimal diagnostic performance either as individual biomarkers or in combination does not favor their use for triage testing.

## 1 Introduction

Tuberculosis (TB) is the leading cause of death from a single infectious agent, with an estimated 1.6 million deaths and 10.6 million TB cases reported in 2021 (1). In the past decade, molecular methods based on real-time polymerase chain reaction (PCR) for TB diagnosis, such as the Xpert MTB/RIF (Cepheid, Sunnyvale, CA, USA), have been widely used worldwide (2, 3). However, the need for stable electricity and their costs have made it difficult to establish it as a routinely used test, especially in low-income countries. In settings with a high TB incidence, large numbers of patients with symptoms suggestive of TB undergo testing for diagnosis, resulting in large laboratory workloads. Most of the current TB diagnostics are sputum-based; thus, a point-of-care non-sputum-based triage test for identifying patients who need further testing or for detecting TB by identifying the presence of biomarkers or biosignatures is a priority (4).

Serum levels of acute-phase proteins and cytokines are increased in individuals with overt TB (5). A systematic review and meta-analysis indicated that several markers such as C-reactive protein (CRP), α1-acid glycoprotein (AGP), and interferon-gamma-inducible protein 10 (IP-10) could be used for screening as triage tests to determine who should undergo further confirmatory tests (6). CRP concentrations vary rapidly as an innate response to injury, local inflammation, and infection. AGP, usually present in low amounts in plasma, increases during the acute-phase response to infections. IP-10, a chemokine mainly expressed by antigen-presenting cells, is induced by innate and adaptive immunity mechanisms. The meta-analysis indicated that these three markers have good potential as screening tests (6), but the number of studies was limited, and further studies were needed to strengthen the evidence base. In addition, AGP and α1-anti-trypsin (AAT), well-known acute-phase proteins that are upregulated in various inflammatory conditions and disorders, were helpful for predicting an anti-TB treatment response (7), but their usefulness for an active-TB diagnosis should be further explored.

This study aims to determine whether CRP, IP-10, AGP, and AAT could be used as screening tests in patients with presumptive TB in patients who were prospectively screened for TB.

## 2 Material and methods

### 2.1 Study population

This was a prospective study of consecutive adult patients with signs and symptoms of presumptive TB attending the TB diagnostic laboratory at the Institute of Phthisiopneumology “Chiril Draganiuc,” Republic of Moldova. At the time of the study, the TB incidence in the Republic of Moldova was 95 (82–110) per 100,000 inhabitants (8). Patients with clinical symptoms of presumptive TB were enrolled from April to June 2018. The inclusion criteria were the following: adult patients with presumptive TB diagnosis accepting to participate in the study who provided signed written informed consent and blood for the study purpose. The exclusion criteria were those currently receiving anti-TB treatment, those with a history of past active TB, or patients who preferred not to participate and did not provide signed informed consent. Patients were mainly from the city and the area of influence of Chisinau, and they were directly visiting the institute or were referred by district- or municipal-level hospitals and primary care centers. Patients were interviewed to obtain clinical and demographic information and underwent chest x-rays, sputum smear microscopy with Ziehl-Neelsen staining, and liquid culture with a BACTEC MGIT 960 TB system (BD, Franklin Lakes, NJ, USA). Individuals with abnormal chest X-rays were tested using Xpert. Phenotypic drug susceptibility testing was performed with BACTEC MGIT 960 according to routine clinical practice. The TB laboratory of the Institute of Phthisiopneumology “Chiril Draganiuc” is fully equipped with the technology and the expertise needed for performing smear examinations, microbiological culturing (liquid and solid), phenotypical drug susceptibility testing, and molecular methods (Xpert and others).

### 2.2 Biomarker detection

Whole blood was collected by venipuncture and plasma was harvested the same day and stored at −20°C. Plasma samples were shipped to the Institut d'Investigació Germans Trias i Pujol (IGTP; Badalona, Spain), and the LSTM (Liverpool, UK) for biomarker detection. CRP was quantified in blood by a capillary puncture at the Institutul de Ftiziopneumologie “Chiril Draganiuc” (Chisinau, Republic of Moldova) using the semi-quantitative Actim CRP rapid test (Medix Biochemica, Espoo, Finland) and in plasma using the CRP test for the Eurolyser CUBE instrument (Eurolyser Diagnostica GmbH, Salzburg, Austria) at LSTM. Actim CRP provides CRP concentrations as <10 mg/l, 10–40 mg/l, 40–80 mg/l, and >80 mg/l. Biomarkers were quantified in all plasma samples at the IGTP using different ELISA kits: CRP (Human C-Reactive Protein/CRP Quantikine ELISA Kit, R&D Systems, Inc., Minneapolis, USA), IP-10 (Human CXCL10/IP-10 Quantikine ELISA Kit, R&D Systems, Inc., Minneapolis, USA), AGP (Human alpha 1-Acid Glycoprotein Quantikine ELISA Kit, R&D Systems, Inc., Minneapolis, USA), and AAT (Human alpha 1 Antitrypsin ELISA Kit SERPINA1, Abcam, Cambridge, USA).

### 2.3 Statistical analysis

The median and interquartile ranges (IQRs) were calculated for all biomarkers, and differences between study groups were compared using the Mann–Whitney *U* and Kruskal–Wallis tests. P-values less than 0.05 were considered statistically significant. Multiple linear regression analyses were performed to determine the effects of age, gender, and smoking as covariates for the biomarkers. Diagnostic accuracy was estimated using receiver operating characteristic (ROC) curves and by estimating the area under the curve (AUC) to describe the yield. Cutoffs were calculated using the Youden Index, and we checked if any biomarker met the minimum target product profiles for a non-sputum-based triage test, that is, sensitivity above 90% and specificity above 70%. Correlations between biomarkers were calculated using Spearman's rho correlation coefficients (*r*). Comparisons of the semi-quantitative values obtained by the CPR test were calculated using the Fisher test. Analyses were performed using GraphPad Prism 8.4.1 (GraphPad, San Diego, CA, USA) and SPSS version 15.0 (SPSS Inc, Chicago, IL, USA). The staff who performed the detection of the biomarkers were blinded to previous test results and clinical information.

## 3 Results

A total of 301 participants with presumptive TB were enrolled in the Republic of Moldova. Demographic data of the participants are presented in Table 1. Of these, 24 (8%) were excluded (3 [1%] were receiving TB treatment, 8 [3%] had contaminated cultures, and 13 [4%] had their chest x-ray interpreted as images compatible with former TB episodes). The mean age of the 277 participants included in the analysis was 48.7 years, with a standard deviation of 15.0. Of these 277 participants, 164 (59.2%) were men and 153 (55.2%) were smokers. Most smokers (147, 96.1%) were men.

**Table 1:** Demographic characteristics of the study participants.

	**Bacteriologically confirmed tuberculosis (BC TB, *n* = 85)**	**Bacteriologically negative tuberculosis (BN TB; *n* = 40)**	**Not TB (*n* = 152)**	***p*** **-values**		
				**BC TB vs. BN TB**	**BC TB vs. Not TB**	**BN TB vs. not TB**
**Age** ^a^						
Mean ±*SD* (48.7 ± 15.0)	46.0 ± 15.0	40.7 ± 16.0	52.4 ± 13.7	0.07	0.001	<0.001
**Gender** ^b^						
Men (164)	62 (72.9%)	24 (60%)	78 (51.3%)	0.15	0.001	0.33
Women (113)	23 (27.1%)	16 (40%)	74 (48.7%)			
**BMI** ^b^ ^,^ ^c^						
<18.5 (37)	9 (10.5%)	5 (12.5%)	23 (15.1%)	0.50	0.39	0.91
18.5–24.9 (132)	36 (42.5%)	22 (55%)	74 (48.7%)			
25.0–29.9 (77)	28 (32.9%)	9 (22.5%)	40 (26.3%)			
>30 (31)	12 (14.1%)	4 (10%)	15 (9.9%)			
**Smoker** ^b^						
No (124)	24 (28.2%)	20 (50%)	80 (52.6%)	0.02	<0.001	0.77
Yes (153)	61 (71.8%)	20 (50%)	72 (47.4%)			
**Number of tobacco packs/day** ^b^						
1 (106)	45/61 (73.8%)	13/20 (65%)	48/72 (66.7%)	0.11	0.58	0.25
2 (34)	10/61 (16.4%)	7/20 (35%)	17/72 (23.6%)			
3 (13)	6/61 (9.8%)	0 (0%)	7/72 (9.7%)			

SD, standard deviation; BMI, body mass index.

a Student's t-test. The p-value comparing the three groups together by an analysis of variance test is < 0.0001.

b Chi-square test, or Fisher's exact test when a value is <5.

c P = 0.24 by the analysis of variance test.

Eighty-five (30.7%) participants had bacteriologically confirmed TB with positive culture, of which 71 participants had positive Xpert results (Table 1). Of the 85 participants, 5 (5.9%) had extrapulmonary TB. Fifty-six (65.9%) participants with bacteriologically confirmed TB had drug-susceptible TB and 29 (34.1%) had drug-resistant TB. Among the latter group, 4 had isoniazid mono-resistance. Moreover, 24 had multidrug-resistant TB (isoniazid and rifampicin), of which 1 was also resistant to fluoroquinolones and 6 were resistant to kanamycin. One patient had extensively drug-resistant TB. Forty participants (14.4%) were classified as having bacteriologically negative TB based on the clinical history, clinical presentation, and x-ray findings but had negative smear, culture, and Xpert results. Five of them had extrapulmonary TB. In addition, 152 (54.9%) participants were considered to not have TB (patients without TB) based on negative smears and cultures and normal x-rays.

**Figure 1:**
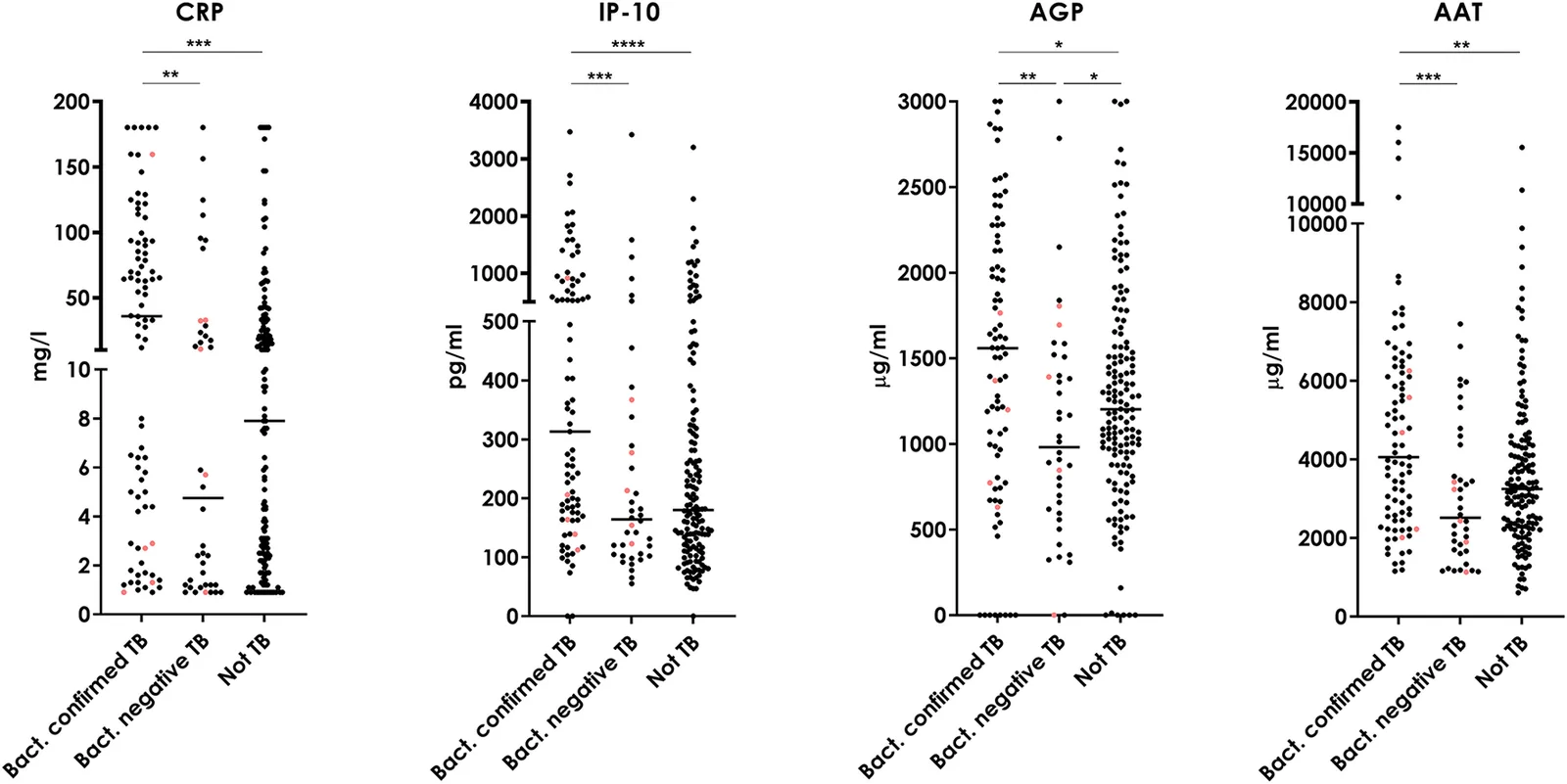
C-reactive protein (CRP), interferon-γ-induced protein 10 (IP-10), α1-acid glycoprotein (AGP), and α1-anti-trypsin (AAT) in patients with bacteriologically confirmed and bacteriologically negative TB or those without TB in the prospective study in Moldova. Pink dots represent cases with extrapulmonary TB. Two-tailed Mann–Whitney *U* test: *p < 0.05; **p < 0.01; ***p < 0.001; ****p < 0.0001. The statistical significance considering all groups together by means of the Kruskal–Wallis test was for CRP, *p* = 0.0002; for IP-10, *p* < 0.0001; for AGP, *p* = 0.0038; and for AAT, *p* = 0.0006.

**Figure 2:**
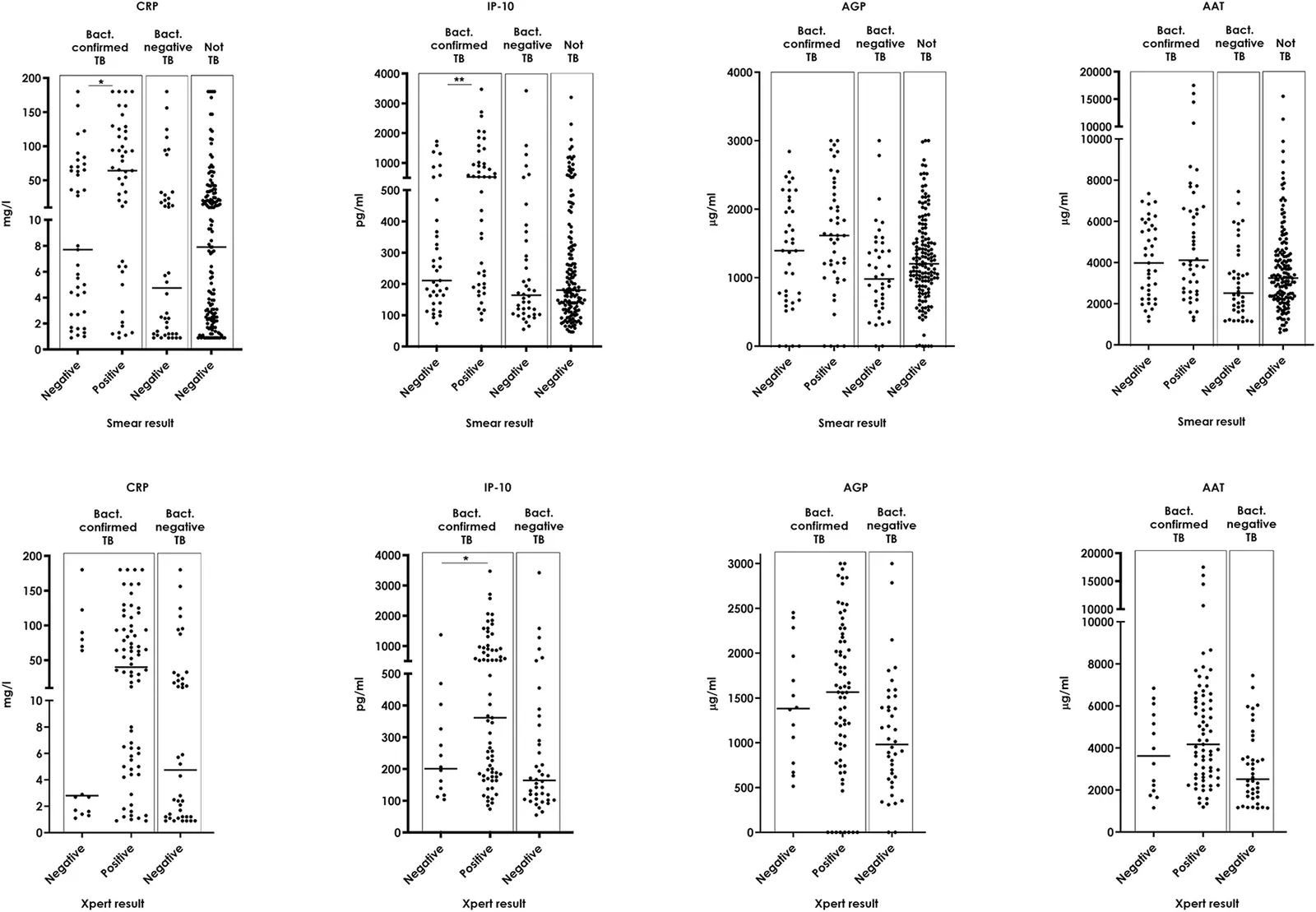
C-reactive protein (CRP), interferon-γ-induced protein 10 (IP-10), α1-acid glycoprotein (AGP), and α1-anti-trypsin (AAT) in patients with bacteriologically confirmed, those with bacteriologically negative, and those without TB by smear microscopy and Xpert results. In patients with bacteriologically confirmed TB: CRP (median [interquartile range]) in patients with positive smear results (64.2 mg/l, 6.4–117.9); CRP in patients with negative results (7.7 mg/l, 2.7–69.7; *p* = 0.02); IP-10 in patients with positive smear results (524.3 pg/ml, 195.7–981.4); IP-10 in patients with negative results (211.0 pg/ml, 139.2–403.4; *p* = 0.005); IP-10 in patients with positive Xpert results (200.9 pg/ml, 116.3–345.5); IP-10 in patients with negative Xpert results (361.4 pg/ml, 178.7–901.7; *p* = 0.03). Two-tailed Mann–Whitney *U* test: *p < 0.05; **p < 0.01.

The overall results of all biomarkers are summarized in Table 2, Figure 1. Statistically significant differences were detected in CRP, IP-10, AGP, and AAT levels, which were higher in patients with bacteriologically confirmed TB than in patients with bacteriologically negative TB and patients without TB, although there was a substantial overlap. Among patients with bacteriologically confirmed TB, CRP and IP-10 levels were higher in patients with positive smear results than in those with negative results (Figure 2). IP-10 levels were also higher in patients with positive Xpert results compared to those with negative results.

**Table 2:**
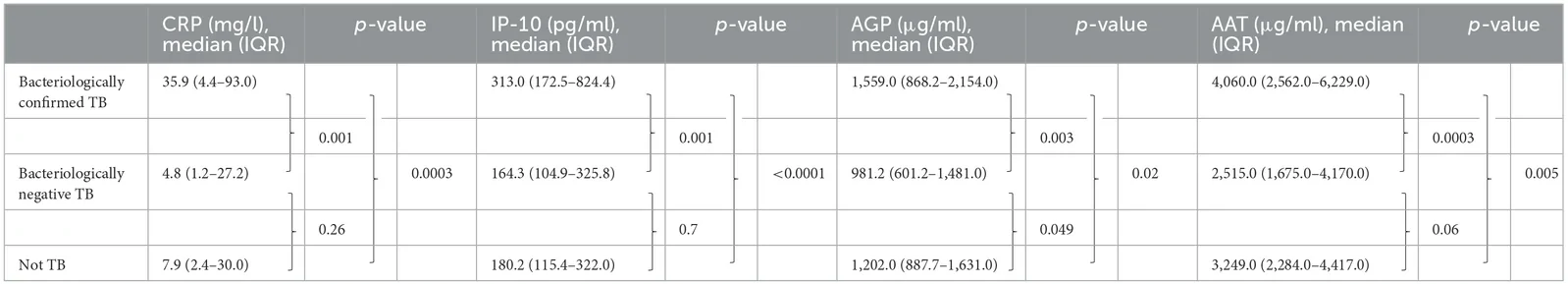
Biomarker concentrations present in plasma samples.

CRP, C-reactive protein; IP-10, interferon-γ-induced protein 10; AGP, α1-acid glycoprotein; AAT, α1-anti-trypsin; IQR, interquartile range; TB, tuberculosis.

Overall, CRP was higher in men (10.3 mg/l, 2.8–42.3 mg/l) than in women (4.6 mg/l, 2.0–18.6 mg/l; *p* = 0.01), in smokers (10.1 mg/l, 2.7–41.1) than in non-smokers (4.8 mg/l, 2.1–20.6 mg/l; *p* = 0.01), and in patients with bacteriologically confirmed (35.9 mg/l, 4.4–93.0 mg/l) than in those with bacteriologically negative TB (4.8 mg/l, 1.2–27.2 mg/l; *p* = 0.001) and those without TB (7.9 mg/l, 2.4–30.0 mg/l; *p* = 0.0003) patients. AGP was higher in men (1,622 μg/ml, 1,082–2,288 μg/ml) than in women (996.1 μg/ml, 587.3–1,765 μg/ml; *p* = 0.02) and in smokers (median 1,668 μg/ml; 1,203–2,301 μg/ml) than in non-smokers (868.2 μg/ml, 606.3–1,587 μg/ml; *p* < 0.001). AGP values were modified by smoking status among patients with bacteriologically confirmed TB (*p* = 0.012). CRP levels were modified by age independent of gender or smoking status in patients with bacteriologically negative TB (*p* = 0.007) and those without TB (*p* = 0.048).

The AUC to differentiate bacteriologically confirmed TB patients from those without TB was <0.7 for all markers (Table 3, Supplementary Figure 1). The correlations (Spearman's *r* < 0.60) between the biomarkers were also low when considering all patients, those with bacteriologically confirmed or bacteriologically negative TB and those without TB. By comparison, when we analyzed the sensitivity and specificity grouping of bacteriologically confirmed and not-confirmed TB as the “TB group,” and the results, in general, showed high sensitivity but very low specificity (Supplementary Table 1).

**Table 3:** Area under the receiver operating characteristic curve for acute phase markers and IP-10 to differentiate those with bacteriologically confirmed tuberculosis from those without tuberculosis.

**AUC (** ***SE*** **) [95% CI]**			
**CRP**	**IP-10**	**AGP**	**AAT**
0.64 (0.039) [0.5665–0.7207]	0.67 (0.037) [0.5931–0.7377]	0.59 (0.041) [0.5093–0.6699]	0.61 (0.039) [0.5342–0.6877]

AUC, area under the receiver operating characteristic curve; SE, standard error; CI, confidence interval; CRP, C-reactive protein; IP-10: interferon-γ-induced protein 10; AGP, α1-acid glycoprotein; AAT, α1-anti-trypsin.

The semi-quantitative CRP test of participants with bacteriologically confirmed, with bacteriologically negative TB, and without TB are shown in Table 4. Among the bacteriologically confirmed TB, a rapid CRP result of 40–80 mg/l and >80 mg/l was obtained for 38 (44.7%) participants. In contrast, among the bacteriologically negative TB, a rapid CRP result of <10 mg/l and 10–40 mg/l was obtained for 33 (82.5%) participants. This difference was statistically significant (*p* = 0.003). Among the patients without TB group, a rapid CRP result of <10 mg/l and 10–40 mg/l was obtained for 121 (79.6%) participants. This value was statistically significant (*p* > 0.0001) in comparison to those obtained by the bacteriologically confirmed group but not significant (*p* = 0.825) in comparison with the bacteriologically negative TB group. Interestingly, grouping both TB groups (bacteriologically confirmed and not confirmed), the results were also significantly different when compared with the patients without TB group (*p* = 0.0045). The CRP results obtained with the semi-quantitative test correlated well with those obtained with the quantitative test, with a kappa (standard error) of 0.736 (0.033; Supplementary Table 2).

**Table 4:** CRP rapid test results obtained in the different groups of patients in blood obtained by capillary puncture.

	**CRP rapid test results**			
	<**10 mg/l**, ***n*** **(%)**	**10–40 mg/l**, ***n*** **(%)**	**40–80 mg/l**, ***n*** **(%)**	>**80 mg/l**, ***n*** **(%)**
Bacteriologically confirmed TB (*n* = 85)	30 (35.3)	17 (20.0)	11 (12.9)	27 (31.8)
Bacteriologically negative TB (*n* = 40)	20 (50.0)	13 (32.5)	0 (0)	7 (17.5)
Not TB (*n* = 152)	73 (48.0)	48 (31.6)	15 (9.9)	16 (10.5)

CRP, C-reactive protein; TB, tuberculosis.

## 4 Discussion

In this study, we evaluated the potential usefulness of CRP, IP-10, AGP, and AAT levels to be used as a TB screening test in patients with presumptive TB disease. In general, the levels of all the biomarkers were higher in patients with TB than in the other groups (*p* < 0.05). ROC curve analyses showed an AUC lower than 0.7 for all the biomarkers, and low correlations (Spearman's *r* < 0.6) were found between biomarkers. Levels of the biomarkers were different between the patient groups, but there was a substantial overlap that limited their diagnostic value for triage testing.

In a recent study performed in a cohort of 332 children, Jaganath et al. (9) observed that the CRP was unable to achieve the accuracy targets for a TB triage test. In the present study, CRP levels were higher in patients with bacteriologically confirmed TB than in those with bacteriologically negative TB and without TB. In a previous systematic review and meta-analysis, CRP >10 mg/l had a sensitivity and specificity of 89% and 57%, respectively, for diagnosing TB (6). In the present study, CRP >10 mg/l had a sensitivity of 58.2% and 42.5% for detecting bacteriologically confirmed TB and bacteriologically negative TB, respectively, and a specificity of 55.9% while considering the patients without TB cases. However, by increasing the cutoff to 40 mg/l, the sensitivity was 44.7%, and the specificity reached 88%, substantially. It is interesting to mention that these results have been obtained using rapid lateral flow assay, which has the potential to play a role in a “one-stop” diagnostics or screening process. This type of technology does not need equipment or electricity and can be easily implemented in low-resource laboratories. In addition, the use of blood by capillary puncture facilitates the obtention of samples, even in peripheral areas.

Notably, in the previous meta-analysis, CRP sensitivity and specificity were higher in people coinfected with HIV-TB (91% and 61%, respectively) than in HIV-uninfected patients (81% and 32%, respectively) (6). In the present study, all patients were HIV-uninfected. Median CRP levels were higher in the smear-positive patients than in the smear-negative ones. This was previously reported in people coinfected with HIV-TB (10) and in people without HIV infection (11), where the CRP levels were also higher in very positive smears as compared to the smears with a scant number of bacilli. This may be due to a higher inflammation state in patients with higher bacillary load.

In the present study, IP-10 levels were higher in patients with bacteriologically confirmed TB than in those with bacteriologically negative TB and without TB. In a previous systematic review and meta-analysis, IP-10 was shown to have a sensitivity and specificity of 85% and 63%, respectively, for TB diagnosis (6). Jacobs et al. reported that IP-10 levels in unstimulated plasma were higher in patients with TB than in those with other respiratory diseases (randomly selected from a biobank of samples of a range of other diagnoses, including viral and bacterial upper and lower respiratory tract infections and acute exacerbations of chronic obstructive pulmonary disease or asthma), and the sensitivity and specificity of IP-10 to diagnose TB were 86% and 73%, respectively (12). In a previous study, the authors tested a large number of biomarkers; however, the optimal diagnostic biosignature included six, none of them being IP-10 (12). IP-10 levels have also been associated with mycobacterial load, according to the semi-quantitative Xpert results (13). In the present study, IP-10 levels were higher in patients with Xpert-positive results than those with negative results. This was also previously detected among people living with HIV (10, 13).

Regarding AGP, in our study, AGP levels were highest in patients with bacteriologically confirmed TB, followed by patients without TB, and by patients with bacteriologically negative TB. This result is justified by the fact that the patients without TB are not healthy controls; they are patients with respiratory symptoms that were studied for presumptive TB. Asseo et al. reported that, in patients with pleural effusion, AGP levels in serum were higher in patients with active TB than in those with malignant disease and with non-malignant noninflammatory disease, but there was an overlap of AGP levels between the three groups of patients (14). Furthermore, Fassbender et al. showed that AGP levels in serum were higher in patients with active TB and bacterial pneumonia than in healthy controls but did not differ between the former (15). Note that AGP is a heterogeneous population of glycoforms, with the same protein sequence but different oligosaccharide chains. The analysis of the AGP glycosylation pattern yielded a coefficient that differentiated between active TB and bacterial pneumonia with a sensitivity and specificity of 86% and 93%, respectively (15). In all study groups, the coefficient did not correlate with AGP concentrations, confirming the independence between glycosylation and AGP serum levels (15). Finally, Immanuel et al. reported that AGP levels were higher in patients with different types of active TB (pulmonary TB, abdominal TB, and children with TB meningitis) than in healthy volunteers (16). Recently, Kang et al. (17), in a study looking for biomarkers able to distinguish latent from active TB patients, reported that AGP was significantly increased (*p* > 0.05) in active TB cases.

Regarding AAT, in the present study, AAT levels were higher in patients with bacteriologically confirmed TB than in those with bacteriologically negative TB and without TB. Several studies have reported that AAT levels were higher in patients with TB than in control individuals (7, 18–21). However, despite the differences in the levels of this biomarker, it was not possible to distinguish patients with active TB from other pulmonary diseases with good diagnostic performance.

The use of biomarkers could improve the current screening strategies to reduce the number of Xpert tests to be performed. In a study with people coinfected with HIV-TB, where the routine case-finding algorithm consisted of symptom screening followed by Xpert testing, the addition of CRP as a screening test was evaluated. CRP screening halved the proportion of patients requiring Xpert testing and detected a lower proportion of TB cases (81% vs. 100%) among patients engaged in care (with a previous HIV clinic visit) but a similar proportion (93% vs. 98%) among patients that were new to HIV care (22). In the present prospective study, the use of CRP in combination with IP-10, AGP, and AAT yielded suboptimal sensitivity and specificity for distinguishing cases with bacteriologically confirmed TB from cases without TB, and both the positive and negative predictive values were low and thus did not favor the use of these biomarkers.

A biosignature that met the World Health Organization performance criteria for a triage test (4) consisted of six markers—SYWC (an IFN-γ-inducible Trp-tRNA-synthetase), kallistatin, complement C9, gelsolin, testican-2, and aldolase C-. This biosignature was identified in a large biomarker screening study by proteomic analysis in serum samples from patients with presumptive active pulmonary TB from seven high-incidence countries (23). In the present study, the number of biomarkers tested was limited, and other biomarkers such as procalcitonin or neopterin may be also useful (24). The biomarker levels might also be affected by other factors, such as comorbidities, especially HIV status, and medication taken by the patients. In addition, an analysis of the biomarkers during treatment could have added value to the performance. It has been previously shown that CRP levels were found to decrease during TB treatment and correlate with the clinical response (5, 25). IP-10 may also have the potential to monitor responses to TB treatment, as IP-10 levels decreased from baseline to month 6 of treatment in stimulated and unstimulated plasma (12, 13, 26) and in urine (27, 28). AGP levels have also been reported to decrease during treatment (7, 16), as well as AAT levels (7, 12, 29).

In conclusion, the levels of the tested biomarkers were different throughout the patient groups included. Although there was substantial overlapping of values among the different study groups, resulting in suboptimal diagnostic performance, promising results with the semi-quantitative test for CRP were obtained in favor of their use in triage testing. The inclusion of the new tests based on non-sputum samples could improve the screening algorithms, reducing the number of Xpert tests to be performed. In addition, the detection of biomarkers (such as the CRP) using point-of-care tests, could improve TB diagnostic in peripheral areas with low-resource laboratories. In the present times, diagnosing TB is still particularly challenging, especially for some vulnerable populations. In this context, the evaluation of combinations of current and newer TB tests, including biomarkers such as those assessed in this study, can facilitate TB diagnosis in the locations where they are not currently available, increasing the number of patients correctly diagnosed, increasing access to the treatment, and reducing mortality and TB transmission. Further future research in this approach should be developed urgently in the coming years.

## Data availability statement

The raw data supporting the conclusions of this article will be made available by the authors, without undue reservation.

## Ethics statement

Ethical approval was obtained from the Research Ethics Committees of the Liverpool School of Tropical Medicine (LSTM, UK), the Institute of Phthisiopneumology “Chiril Draganiuc” (Republic of Moldova), and the Hospital Universitari Germans Trias i Pujol (HUGTP, Spain; reference number CEI_PI-16-051). Written informed consent was obtained from all participants.

## Author contributions

BM-Moy: Formal analysis, Methodology, Writing—original draft. RV-H: Formal analysis, Methodology, Writing—original draft. NC: Methodology, Investigation, Writing—review & editing. BM-Mor: Methodology, Investigation, Writing—review & editing. AL: Methodology, Investigation, Writing—review & editing. AC: Methodology, Investigation, Writing—review & editing. IL: Performed analysis, Writing—review & editing. DS: Performed analysis, Writing—review & editing. CP-A: Methodology, Investigation, Writing—review & editing. VC: Methodology, Investigation, Writing—review & editing. KK: Methodology, Investigation, Writing—review & editing. LC: Methodology, Supervision, Writing—original draft. JD: Methodology, Supervision, Writing—original draft.
