# Supplementary material for: Acute phase proteins and IP-10 in plasma for tuberculosis diagnosis
Source: Front Tuberc. 2023 Nov 29;1:1267221. doi: 10.3389/ftubr.2023.1267221 (PMC13494927; doi:10.3389/ftubr.2023.1267221)
Supplement: Supplementary file 1 [file Table_1.pdf]

Supplementary Material

1 Supplementary Table

Supplementary Table 1. Cut-offs of the biomarkers for a screening test (sensitivity >90%), and their sensitivity and specificity for distinguishing cases with bacteriologically confirmed or bacteriologically negative TB from cases with no TB, and for distinguishing cases with bacteriologically confirmed TB from cases with bacteriologically negative TB or no TB.

		Cut-off	Sensitivity (95% CI)	Specificity (95% CI)
BC TB + BN TB vs No TB	CRP	> 1.050	92.7% (86.8- 96.1)	9.9% (6.1- 15.6)
	IP-10	> 98.66	90.4% (84.0-94.4)	19.1% (13.6-26.1)
	AGP	> 331.8	90.4% (84.0-94.4)	4.6% (2.2-9.2)
	AAT	> 1568	90.4% (84.0-94.4)	9.9% (6.1-15.6)
BC TB vs BN + No TB	CRP	> 1.250	92.9% (85.3-96.7)	17.2% (12.5-23.2)
	IP-10	> 110.9	90.6% (82.5-95.2)	24.5% (18.9-31.0)
	AGP	> 457.8	90.6% (82.5-95.2)	9.4% (6.0-14.3)
	AAT	> 1958	90.6% (82.5-95.2)	20.3% (15.2-26.6)
BC TB vs No TB	CRP	> 1.250	92.9% (85.1-97.3)	13.8% (8.8-20.3)
	IP-10	> 110.9	90.6% (82.3-95.9)	23.7% (17.2-31.3)
	AGP	> 457.8	90.6% (82.3-95.9)	7.2% (3.7-12.6)
	AAT	> 1958	90.6% (82.3-95.9)	16.5% (10.9-23.3)

BC TB: bacteriologically confirmed TB; BN TB: bacteriologically negative TB
