# Supplementary material for: Acute phase proteins and IP-10 in plasma for tuberculosis diagnosis
Source: Front Tuberc. 2023 Nov 29;1:1267221. doi: 10.3389/ftubr.2023.1267221 (PMC13494927; doi:10.3389/ftubr.2023.1267221)
Supplement: Supplementary file 2 [file Table_2.pdf]

Supplementary Material

1 Supplementary Table

Supplementary Table 2. CRP test results obtained in relation with the rapid CRP Eurolysed results.

	**CRP Rapid test results**			
**CRP Eurolyser results grouped**	**<10 mg/l [n (%)]**	**10-40 mg/l [n (%)]**	**40-80 mg/l [n (%)]**	**>80 mg/l [n (%)]**
<10 mg/l (n=142)	120 (84.5)	22 (15.5)	0 (0)	0 (0)
10-40 mg/l (n=56)	3 (5.4)	48 (85.7)	5 (8.9)	0 (0)
40-80 mg/l (n=31)	0 (0)	7 (22.6)	17 (54.8)	7 (22.6)
>80 mg/l (n=47)	0 (0)	1 (2.1)	4 (8.5)	42 (89.4)
Total (n=276*)	123 (44.6)	78 (28.3)	26 (9.4)	49 (17.8)

* CRP Eurolyser result was missing for one sample.

kappa (SE) = 0.736 (0.033)
